# Towards Integrated Air Pollution Monitoring and Health Impact Assessment Using Federated Learning: A Systematic Review

**DOI:** 10.3389/fpubh.2022.851553

**Published:** 2022-05-19

**Authors:** En Xin Neo, Khairunnisa Hasikin, Mohd Istajib Mokhtar, Khin Wee Lai, Muhammad Mokhzaini Azizan, Sarah Abdul Razak, Hanee Farzana Hizaddin

**Affiliations:** ^1^Department of Biomedical Engineering, Faculty of Engineering, Universiti Malaya, Kuala Lumpur, Malaysia; ^2^Center of Image and Signal Processing (CISIP), Faculty of Engineering, Universiti Malaya, Kuala Lumpur, Malaysia; ^3^Department of Science and Technology Studies, Faculty of Science, Universiti Malaya, Kuala Lumpur, Malaysia; ^4^Department of Electrical and Electronic Engineering, Faculty of Engineering and Built Environment, Universiti Sains Islam Malaysia, Nilai, Malaysia; ^5^Institute of Biological Sciences, Faculty of Science, Universiti Malaya, Kuala Lumpur, Malaysia; ^6^Department of Chemical Engineering, Faculty of Engineering, Universiti Malaya, Kuala Lumpur, Malaysia

**Keywords:** federated learning, health hazard, deep learning, machine learning, air pollution

## Abstract

Environmental issues such as environmental pollutions and climate change are the impacts of globalization and become debatable issues among academics and industry key players. One of the environmental issues which is air pollution has been catching attention among industrialists, researchers, and communities around the world. However, it has always neglected until the impacts on human health become worse, and at times, irreversible. Human exposure to air pollutant such as particulate matters, sulfur dioxide, ozone and carbon monoxide contributed to adverse health hazards which result in respiratory diseases, cardiorespiratory diseases, cancers, and worst, can lead to death. This has led to a spike increase of hospitalization and emergency department visits especially at areas with worse pollution cases that seriously impacting human life and health. To address this alarming issue, a predictive model of air pollution is crucial in assessing the impacts of health due to air pollution. It is also critical in predicting the air quality index when assessing the risk contributed by air pollutant exposure. Hence, this systemic review explores the existing studies on anticipating air quality impact to human health using the advancement of Artificial Intelligence (AI). From the extensive review, we highlighted research gaps in this field that are worth to inquire. Our study proposes to develop an AI-based integrated environmental and health impact assessment system using federated learning. This is specifically aims to identify the association of health impact and pollution based on socio-economic activities and predict the Air Quality Index (AQI) for impact assessment. The output of the system will be utilized for hospitals and healthcare services management and planning. The proposed solution is expected to accommodate the needs of the critical and prioritization of sensitive group of publics during pollution seasons. Our finding will bring positive impacts to the society in terms of improved healthcare services quality, environmental and health sustainability. The findings are beneficial to local authorities either in healthcare or environmental monitoring institutions especially in the developing countries.

## Introduction

In today's globalization era, inhaling clean air has now become opulent. Environmental issues such as pollutions and climate change worsen due to the impacts of globalization. Air pollution refers to the environmental contamination that occurs either indoor or outdoor caused by any chemical, physical or biological agent that could change the natural characteristics of the atmosphere. According to World Health Organization (WHO), the impacts of ambient air pollution exposure which caused an estimated 4.2 million deaths annually have led to stroke, heart diseases, lung cancer, acute and chronic respiratory diseases (1). Meanwhile, WHO also reported that in the developing countries, one of the sources of indoor pollution are actually coming from household activities such as cooking. It has also become one of the leading causes of diseases such as respiratory diseases and premature death (1).

Pollutants such as particulate matter (PM_2.5_ and PM_10_), ozone (O_3_), nitrogen dioxide (NO_2_), etc. caused the ambient air quality to be below the healthy and normal limit (1). Other pollutants namely polycyclic aromatic hydrocarbons (PAHs) and volatile organic compounds (VOCs) also contributed to ambient air pollution. On the other hand, the indoor air pollution is often contributed by exposure to PM2.5, smoke from tobacco and other sources, heating by burning substances namely coal and kerosene. Exposure to high levels of pollutants whether short-term or long-term contributes to a variety of adverse health outcomes. In this study, the ambient air pollutants and their impacts on health are keen to be explored.

Poor air quality has resulted from the high-level air pollution. To assess the air quality, it is important to monitor the air pollution exposure, and its potential health hazards to human. Hence, a robust predictive model for air quality monitoring is needed to anticipate risk factor due to air pollutant exposure. Recently, researchers have done significant studies on forecasting air pollution, understanding its interaction and associations with other pollutants. Myriad research have been conducted to understand the impacts of these pollutants on health (2–24). Air pollution prediction requires significant information on its contaminants, their sources and interaction with other particles. The outcome of the predictive model is crucial in assisting public to monitor their health conditions and aiding the healthcare management in providing an effective and comprehensive medical service as well as resources planning. In addition, lacking significant studies on air pollution prediction based on socioeconomic activities and forecasting of healthcare services during severe air pollution is still lacking. The following research questions were developed in aiding the process of developing this systemic review:

1. How are the significant pollutants that cause ambient air pollution can be characterized based on the socio-economic, location-specific and its significant associations to the health hazard?
2. What are the health hazards and potential diseases contributed by the pollutant?
3. How do the impacts of pollution contribute to healthcare services and resources planning?

The identification of the pollutants can assist in determining the pollution sources; hence, a mitigation plan can be proposed to reduce the risk of air pollution to human health. In addition, we reviewed the feasibility of predicting air pollution and monitoring air quality using artificial intelligence (AI) and/or machine learning (ML) techniques using pollutants' parameters based on the socio-economic activities, revealing the gap and the novelty of the study. The identified pollutants will be contributed to the air quality policies and management development, aiding the government in monitoring the air quality. Currently, there are limited studies that contributed to the aspects mentioned above. Therefore, by undertaking the systemic review of previous studies, this study aimed to identify the significant pollutants and the sources based on socio-economic activities, and subsequently identify the significant association with the health hazard.

## Materials and Methods

### Literature Search

The systematic review was completed by referring to the standard namely Preferred Reporting Items for Systematic Reviews and Meta-Analyses (PRISMA). PRISMA is performed in evaluating and analyzing related articles on air pollutions from the academic databases. The PRISMA process involves the searching using keywords and article selection and screening. In addition, the processes sorting of inclusion and exclusion criteria, data extraction and synthesis were performed thoroughly for relevant studies and the included studies were examined to fit the objective of systemic review in the subject field. The process of the PRISMA will be further explained in section 2.2 and section 2.3.

### Resources

The relevant literature of this systematic review related to air pollution prediction was found from the two major databases, namely Scopus and Web of Science where both covered a variety of study fields. The study fields that are included in both databases are engineering, environmental research, artificial intelligence and health science. In this review study, a few additional databases were added to enhance the comprehensiveness and the possibility of searching the relevant articles of the study subject area. The additional selected databases are Science Direct, IEEE Xplore, Emerald, SAGE Journal and Dimensions. The systematic review of the articles was performed from the year of 2010 to 2022 to ensure only relevant and current state-of-the-art technologies are included in the study.

### Article Selection

Article selection explains how the articles were selected based on PRISMA guideline. It involved three steps of identification, screening, and eligibility of the articles to be included in the study. The selection of articles was achieved by using the search string keywords of databases as shown in Table 1 and identify the relevant articles through the inclusion and exclusion criterion. The articles were then undergone further screening through the title, keywords and abstract of the articles. Relevant papers were also selected by the field of research such as engineering, environmental science, artificial intelligence and health science. Lastly, it will be screened for the eligibility of the article by revieing the full-text of the articles.

**Table 1:** Search string of databases (Scopus and Web of Science).

**Database**	**Search string**
Scopus	(TITLE-ABS-KEY (“*air quality”*) AND TITLE-ABS-KEY (“*machine learning”*) AND TITLE-ABS-KEY (“*health”*))
Web of science	(“*air quality”*) AND TITLE-ABS-KEY (“*machine learning”*) AND TITLE-ABS-KEY (“*health”*)

#### Identification

The identification and selection of articles relevant to the study begin with the selection of keywords of the subject area. Appropriate keywords are crucial to achieve only the relevant articles accurately. Often, in constructing the appropriate keywords, thesaurus and previous research were referred to and considered. Once the keywords have been constructed, the search strings were developed for advanced searching. Table 1 illustrates the search string of databases (Scopus and Web of Science). In this stage, data analysis is performed to analyze and summarize the content of the selected articles by extracting important techniques, parameters, and deliverables. The is crucial to identify the scientific research gap in this research. The outcomes of the analysis will be discussed in the section of results and discussion.

Moreover, during retrieving the articles from the databases, criteria such as inclusion and exclusion should be considered for appropriate searching, minimizing the chance of retrieving irrelevant articles. Setting the criteria were significant to narrow the searching of subject areas for the latest research relevant to the study. Table 2 described the inclusion and exclusion criteria that applied in advanced search of the databases. The journal type and timeline are limited to research article within 2010 to 2022, to ensure the articles and resources content are comprehensive and up to date. This is crucial to produce a more specific research and optimized outcomes. From the keywords searching, 2,417 articles from Scopus and 265 articles from Web of Science were retrieved from the search. In addition, from the additional databases, there were a total of 14,442 articles retrieved from the other 5 databases, namely Science Direct, SAGE Journal, Emerald, IEEE Xplore and Dimensions as shown in Table 3. Concurrently, other methods such as searching from websites, organizations and citations were carried out to identify relevant studies. From this method, 86 references were identified using the same keywords. In total, 17,210 references were identified including articles and reports via database and other methods.

**Table 2:** Inclusion and exclusion criteria for the searching in databases.

**Criterion**	**Inclusion**	**Exclusion**
Literature type	Journal (research article)	Journal (review articles), conference proceeding, book series, book chapter, book, encyclopedia
Language	English	Non-English
Timeline	2010–2021	< 2010
Area	Engineering, Environmental Science, Health Science, Artificial Intelligence	Other than Engineering, Environmental Science, Health Science, Artificial Intelligence

**Table 3:** Search strings for 7 databases.

**Searching texts**	**Science** **direct**	**IEEE** **Xplore**	**Web of** **science**	**SAGE**	**Emerald**	**Dimensions**	**Scopus**
Air pollution AND telehealth	12	0	7	0	0	395	50
Air quality AND telehealth	11	0	2	2	0	335	41
Air pollution AND digital health	9	0	6	0	10	364	2
Air quality AND digital health	10	0	0	1	9	310	71
Sustainable health AND Air quality	51	0	1	1	8	282	77
Sustainable health AND Air pollution	35	0	7	1	6	350	82
Air quality AND Machine learning AND Health	1407	14	168	18	60	7390	2000
Air quality AND Deep Learning AND Health	451	8	74	5	23	2864	94
Total including duplicates	1986	22	265	28	116	12290	2417
Sub-total including duplicates	17124						
Total selected	18						

#### Equations Screening

**Figure 1:**
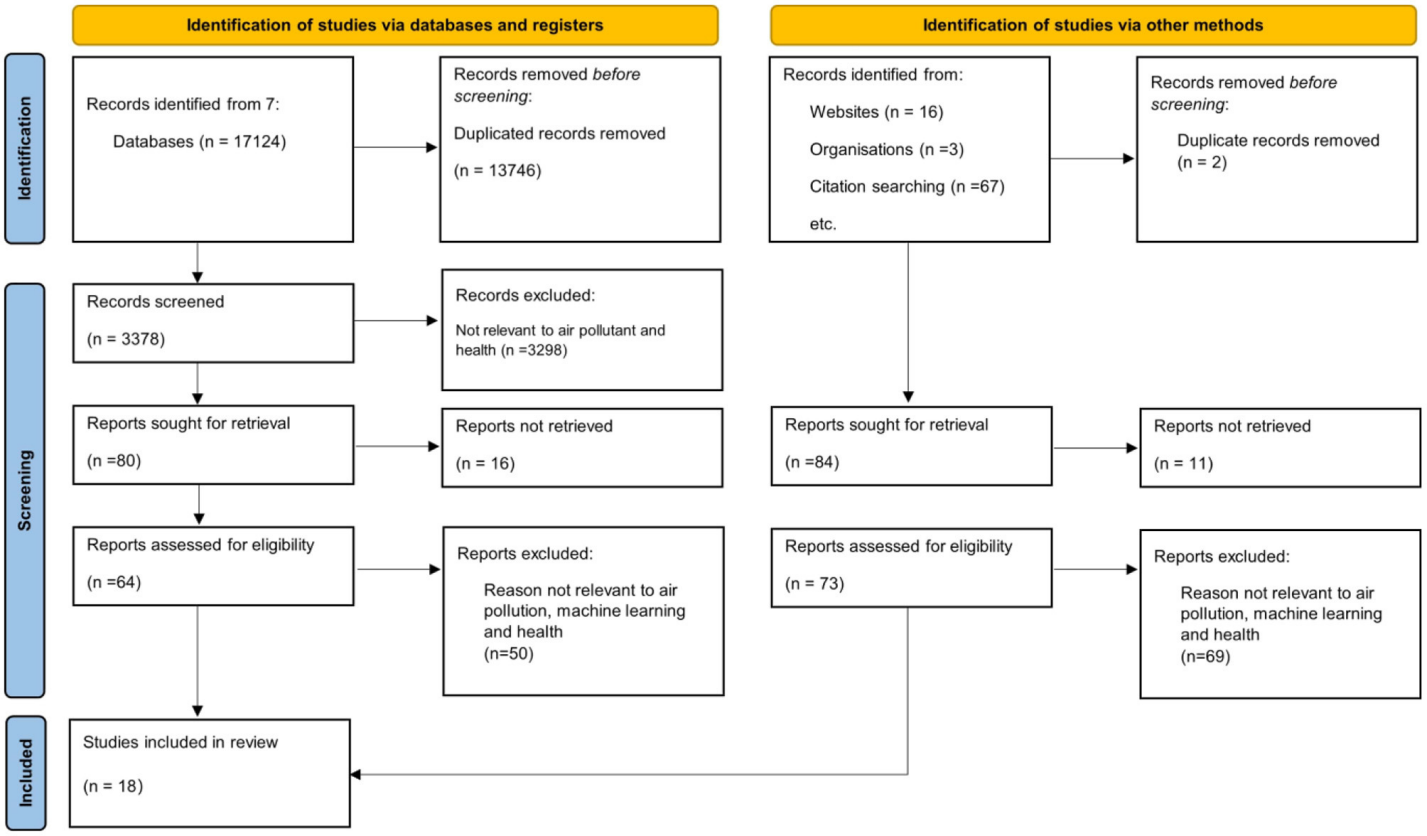
PRISMA flow chart of the review adopted from the PRISMA 2020 statement: an updated guideline for reporting systematic reviews (25).

From the 17,210 references, 17,124 references were retrieved via databases and registers and 86 references were identified via databases and other methods as shown in Figure 1. The references retrieved were divided into two categories namely duplicated reference and irrelevant subject fields. From this process, 13,746 articles were found to be duplicated in the databases and 2 references in the other methods. After the duplicated references were removed, the number of remaining articles were 3,378 and 84 for databases and other methods respectively. After the duplicated references were removed, the remaining references were further screened, by examining the title, keywords and abstract thoroughly. The terms involved were air pollution or air quality and machine learning techniques in the title and keywords.

On the other hand, in the abstract, artificial intelligence or machine learning techniques as well as the impact of air pollution on health were depicted. From the 3,378 articles from databases, 3,298 articles were excluded due to their irrelevant content to the subject areas, resulting in 80 articles that were relevant to the study. However, 16 out of 80 articles were excluded due to unretrievable full-text articles from databases and 11 out of 84 from other methods. Hence, there were only 64 articles from databases and 73 reports were found via other methods. These references were chosen for the further steps of article selections.

#### Eligibility

Eligibility screening filters the references by reviewing the full text of the articles to ensure the contents is eligible for the study. From the previous steps, full text of 64 articles and 73 references were involved in reviewing the eligibility. The contents of these articles such as the objective, methodology, input features, research outcomes, research contributions were reviewed and examined to ensure the inclusion and exclusion criteria were fulfilled. As a result, only 13 articles were selected from the database and 4 references were selected from other methods. 50 articles and 69 references were excluded from the screening on eligibility due to their irrelevant content. They were not implementing machine learning techniques on predicting the air pollution and irrelevant to the health hazards caused by air pollution. Thus, the total number of articles selected for this study was 18, as illustrated in Figure 1.

#### Quality Assessment and Data Extraction

The quality assessment of the articles selected was performed by the authors to ensure the usefulness of the articles to the research. The articles were assessed through the elements such as research aim, research methodology, contribution and highlights from the articles. In addition, the information extracted from the articles was performed for further synthesis and analysis by the authors. The information was compiled in a well-organized summary table. With the tabulation of the information, the crucial parameters and features were extracted and examined thoroughly among the authors.

## Results

In this section, the results of the article reviews will be discussed. The general outcomes and contributions from the reviewed articles will be elaborated in the sub-section of background and studies finding. The similarities and research gaps identified from the reviews will be illustrated in a table under the sub-section of the main findings. The analysis is crucial in determining the novelty and the worthiness of the search direction.

### Main Studies Findings

The analysis involved 18 articles in the study. From the analysis, we can conclude that the studies showed the significant impacts of air pollution on the health of different regions worldwide. Health hazards identified from the studies were categorized into 3 categories: i) cardiorespiratory diseases, ii) premature and birth death and iii) cancers. In addition, from the analysis, the pollution markers were identified which significantly contributed to the health hazard. The pollution markers were particulate matters (PM), ozone (O_3_), carbon oxide (CO), nitrogen dioxide (NO_2_), sulfur dioxide (SO_2_), and other suspended materials such as volatile organic compounds (26).

According to WHO, these contaminants were potentially harmful to human health as well as to the environment and ecosystem. Table 4 shows the summary of the general overview of the findings from the review.

**Table 4:** Summary and overview of the review findings.

**Authors**	**Air pollution markers**	**Health hazard impact from air pollution**	**Techniques**	
			**Prediction/ monitoring model**	**Association assessment**
Reid et al. (11) *(United States (US))*	Particulate Matter (PM_2.5_), Ozone (O_3_)	Respiratory diseases	Generalized additive model (GAM), generalized boosting model (GBM), k-nearest neighbor model regression, lasso regression	Poisson generalized estimating equations
Usmani et al. (15) *(Malaysia)*	Particulate Matters (PM_10_), Ozone (O_3_), carbon monoxide (CO), nitrogen oxides (NO_x_), nitrogen dioxides (NO_2_), nitrogen monoxide (NO), sulfur dioxide (SO_2_)	Cardiorespiratory diseases	Enhanced long short-term memory (ELSTM)	-
Tusnio et al. (14) *(Poland)*	Sulfur dioxide (SO_2_), nitrogen dioxides (NO_2_), nitrogen oxides (NO_x_), carbon monoxide (CO),Ozone (O_3_), Particulate Matters (PM_2.5_,PM_10_), benzene (C_6_H_6_), Lead (Pb), Arsenic (As), Cadmium (Cd), Nickel (Ni), Benzo(a)pyrene (BaP) in PM_10_ size	Various types of cancers	Random forest	Pearson correlation coefficient
Wang et al. (17) *(China)*	Particulate Matters (PM_2.5_, and PM_1_)	Blood cell counts for pregnancy preparation	-	Generalized additive mixed model (GAMM)
Achebak et al. (2) *(Spanish)*	Ozone (O_3_), nitrogen monoxide (NO)	Premature mortality	-	Quasi-Poison regression model
Wang et al. (16) *(China)*	Particulate Matters (PM_2.5_, and PM_1_)	Blood pressure	Random forest model	Generalized additive mixed model (GAMM)
Zani et al. (4) *(Equatorial Asia)*	Particulate Matters (PM_2.5_, and PM_10_)	Premature mortality	Deep neural network (DNN)	Generalized exposure mortality mixed model
Zou et al. (18) (*Western U.S, Pacific Northwest (PNW))*	Particulate Matters (PM_2.5_)	Mortality	Ordinary multi-linear regression, generalized boosting method, random forest	-
Cazzolla Gatti et al. (6) *(Italy)*	Particulate Matters (PM_2.5_ and PM_10_), nitrogen dioxide (NO_2_), sulfur dioxide (SO_2_), carbon monoxide (CO), Benzene (C6H6), ozone (O_3_)	Mortality and infectivity of COVID-19	Random forest regression, Pearson's correlation coefficient	-
Sethi et al. (12) *(India, Delhi)*	Carbon monoxide (CO), sulfur dioxide (SO_2_), particulate matters (PM_2.5_), Ozone (O_3_), nitrogen dioxide (NO_2_), ammonia (NH_3_), toluene (C_7_H_8_), benzene (C_6_H_6_)	Covid-19 fatalities	Decision tree, linear regression, random forest	-
Peng et al. (10) *(China)*	Carbon monoxide (CO), sulfur dioxide (SO_2_), nitrogen dioxide (NO_2_), ozone (O_3_), particulate matters (PM _ <2.5_)	Respiratory disease	Bagging, adaptive boosting, and random forest	-
Shen et al. (13) *(South Korea)*	Particulate matter (PM_2.5_ and PM_10_), carbon monoxide (CO), nitrogen dioxide (NO_2_), sulfur dioxide (SO_2_)	-	Prophet forecasting model (PFM)	-
Al Noaimi et al. (3) (*Lebanese Republic)*	Particulate Matters (PM_2.5_), sulfur dioxide (SO_2_), nitrogen dioxide (NO_2_)	Prenatal and birth defect	Multivariate regression models	-
Li et al. (9) *(China)*	Particulate Matters (PM_2.5_)	Esophageal cancer	-	quasi-Poisson generalized linear model
Amoroso et al. (27) (*European Countries)*	carbon monoxide (CO), nitrogen dioxide (NO_2_), ozone (O_3_), methane (CH_4_), formaldehyde (CH_2_O), aerosol	Covid-19 mortality	Random forest	-
Hadei et al. (7) *(Iran)*	Particulate Matters (PM_2.5_ and PM_10_), nitrogen dioxide (NO_2_), ozone (O_3_)	Covid-19 mortality and morbidity	Distributed-lag non-linear model (DLNM), generalized additive model (GAM)	-
Li et al. (8) *(China)*	Particulate Matters (PM_2.5_)	Esophageal cancer	-	Geographic weighted Poisson Regression
Ren et al. (28) *(China)*	Particulate Matters (PM_10_)	Congenital heart defects	Random forest (RF) and gradient boosting (GB)	-

Based on the review performed, the most significant and dominant pollutant marker was the particulate matter particles (PM_2.5_). The majority of the studies conducted had analyzed the impact of particulate matter (PM_2.5_, PM_10_, PM_ <2.5_) on human health (3, 5–18, 28). Pollution markers such as ozone (O_3_), carbon monoxide (CO), nitrogen dioxide (NO_2_) and sulfur dioxide (SO_2_) are among other important contaminants that were studied by (2–4, 6, 7, 10–15). Chemicals such as nitrogen oxides (NO_x_) also played crucial roles in causing adverse health effects in humans, for instance, cardiorespiratory diseases and cancers.

To identify the health hazard contributed by poor air quality, a predictive monitoring system shall be established to trace the hidden interaction and effects of air pollution on health. Predictive or monitoring systems are often developed using artificial intelligence (AI) techniques such as machine learning and deep learning. Machine learning techniques that are commonly utilized are regression, random forest (RF), multilayer perceptron (MLP), support vector machine (SVM) etc while deep learning techniques involved namely deep neural network (DNN), long short-term memory (LSTM) have emerged as new predictive tool in anticipating air pollutions occurrences. On the other hand, aside from the artificial intelligence-based model, there are some models such as mathematical or statistical models being used to assess the association of air pollutants and health hazards. From the review, a few authors implemented predictive models to estimate the health hazards associated with air pollution. Studies by (5, 10–12, 14, 15, 18) determine the potential relationship and the impact of air pollution to health using the artificial intelligence-based model. Among the authors, there are only studies by (10, 15) that discuss the health hazard hospitalization and emergency department visitation due to air pollution. The techniques used by the authors have enhanced long short-term memory (LSTM), neural network and random forest. By using these techniques, the authors, able to predict the hospitalization of cardiorespiratory and chronic respiratory disease due to long term exposure to pollutants (PMs, ozone, CO, NO, NO_x_, NO_2_, and SO_2_). Meanwhile, studies by (3–6, 12, 14, 16, 18, 28) analyzed the impact of air pollution on health and their association using machine learning techniques such as MLP, random forest, neural network, regression, etc. The contribution of these studies enables to predict and forecast the health hazards contributed by air pollution.

A few other researchers study the association between health hazards and air pollution. To assess the association, researchers developed models using mathematical and statistical knowledge (2, 3, 7, 8, 14, 17). These authors performed the association assessment using modeling of Poison's regression, quasi-Poison distribution, generalized additive model (GAM), etc. These models are useful in helping to identify the association of the health hazards due to pollutants such as PM_2.5_, SO_2_, NO_2_, O_3_, and VOC. From the reviews, often the pollutants mentioned associated with maternal complications and birth defects, premature deaths, mortality and morbidity on Covid-19, cancers, etc. The models are useful in examining the association between the air pollutant and health implications due to air pollution. Hence, prediction of pollution and its impact on health could be performed by developing predictive and monitoring models.

In different studies conducted by Cazzolla Gatti, Velichevskaya (6), Sethi and Mittal (12), Tusnio, Fichna (14), a chemical substance such as benzene (C_6_H_6_) enhanced the effect of air pollution on the human health such as cancers and even increase mortality risk for Covid-19 especially during air pollution. In addition, from the study completed by (12), toluene and ammonia (NH_3_) are important parameters in determining the effect of air pollution on the mortality of Covid-19 during air pollution seasons. On the other hand, (4) have explored a new perspective in predicting chemical compounds such as methane (CH_4_), formaldehyde (CH_2_O), aerosol and their association to Covid-19 mortality during air pollution.

In the study performed by (14), chemical compounds such as benzene (C_6_H_6_), heavy metals such as lead (Pb), Arsenic (As), Cadmium (Cd), and Nickel (Ni) which are in PM10, and aromatic hydrocarbon, benzo-alpha-pyrene (BaP) can caused various types of cancers. From the review, it is proven that these chemical compounds and heavy metals significantly causing cancers in the human body. It is reported that NOx caused intestines and colorectal cancer, PM_2.5_ contributed to lung cancer formation while Arsenic, benzopyrene and nitrogen dioxide trigger large intestine diseases after long-term exposure.

From the review summarized above, we can conclude the health hazard of air pollutions were diverse. From the recent findings of Amoroso et al. (4), Cazzolla Gatti et al. (6), Hadei et al. (7), Sethi et al. (12), air pollution brings impacts on human health as well as to the Covid-19 related hazards. The studies showed that the air pollution affected the mortality and fatalities of the Covid-19 patients, as well as the infected rate or morbidity of Covid-19 with the presence of pollutants in the air. Among the common pollutants extracted from these studies are PM_2.5_, PM_10_, NO_2_, O_3_, and VOC. Besides that, factors such as temperature and meteorological factors are also considered in the studies.

On the other hand, according to Peng et al. (10), Reid et al. (11), Usmani et al. (15), Wang et al. (16) air pollution brings impact to the respiratory system, causing cardiovascular disease. The cardiovascular and respiratory diseases often involved asthma, respiratory infection, chronic obstructive pulmonary diseases (COPD). Besides, air pollution also affects heart rate and blood pressure as mentioned by Wang et al. (16). The study shows that the systolic and diastolic blood pressure increases after experiencing long-term exposure to particulate matter particles. In addition, air pollution exposure tends to increase the visits and admission to the emergency department and eventually admission to the hospital. This can be observed from the study of Peng et al. (10), Usmani et al. (15), which related to the chronic respiratory and cardiorespiratory diseases. It could also lend to fatality for long term effects.

Moreover, the analyses from the review concluded that air pollution hazard to health by causing various types of cancer, namely esophageal cancer, lung cancer, malignant neoplasm, or tumor formation in various parts of the body such as the large and small intestine, etc. Cancer formation occurs often due to long term exposure to harmful pollution markers such as PM_2.5_, NO_2_, and compounds such as arsenic and benzo-alpha-pyrene (BaP) in the dust (14). These harmful components are carcinogenic which bring chronic impacts to the health, and worse, it could result in mortality. Last but not least, from the review, exposure to air pollution could lead to adverse pregnancy outcomes, in both infant and pregnant women proven by Achebak, Petetin et al. (2), Al Noaimi et al. (3), Bruni Zani et al. (5), Wang et al. (17).

According to Al Noaimi et al. (3), exposure to PM_2.5_ during the pregnancy, especially first trimester causes higher risk of birth defect in infants and genitourinary defect in pregnant women, while according to Bruni Zani, Lonati (5) long term impact of PM_2.5_ exposure increases the premature deaths in babies. Aside from PM_2.5_, NO_2_, and O_3_ also contributed to premature mortality, proven by Achebak, Petetin et al. (2). From the study, it is concluded that the premature mortality rate decreases with the NO_2_ reduction. Besides, exposure to air pollutants such as PMs contributes to complications to women preparing for pregnancy and laboring as mentioned in the studies done by Wang et al. (17). In the same research, it was found that white blood cells (WBC), upon long period of exposure to PMs, is greatly reduced and at the same time, red blood cells (RBC) and thrombocytes increased. These turns of events could lead to complications such as immunocompromised due to decrement of WBC and cardiopulmonary diseases (29) such as stroke and myocardial infarction due to increment of RBC (30, 31). In addition, it could also contribute to longer Disability-Adjusted Life Years (DALY) in pregnant women and infants. In addition, maternal exposure to PM_10_ also increase the susceptibility to congenital heart diseases especially during the weeks 3 to 8 in pregnancy, which is the prenatal cardiac development window (28).

### Impact of Air Pollution Markers on Human Health

Previous studies demonstrated the relationships and the association between air pollutants and their impact on health, as well as techniques to predict air pollution events. From the review, we found that the pollution markers vary on the events of accidents. For instance, pollution markers of PM_2.5_ and O_3_ are found in wildfires. This can be supported by the hypothesis of the presence of air pollution markers present in the air are determined by socioeconomic and human activities. Different activities and events produce different air pollutants which lead to various health effects on the human body. Predictive systems such as random forest, long short-term memory and neural network are capable to predict the potential air pollution and its adverse health effect. In the following subsections, we will discuss the health hazards due to pollutants and provide recommendations on the new techniques to predict the health impact due to pollution in future research. There are various pollution markers such as particulate matter particles, ozone, carbon monoxide, nitrogen oxides etc. In this section, we are interested to discuss the formation of pollutants and how do they impact human health.

#### Air Quality Index (AQI)

Air quality index (AQI) refers to an index on the degree of air contamination associated with its health implications which are made available to the public employed by the government. As the AQI grows, so do the public health dangers. The AQI was renamed and amended by the United States Environmental Protection Agency (U.S. EPA) in 1999 from the Pollution Standard Index (33). The AQI varies in all the countries around the world according to the air quality standard established in the country. To assess the air quality level, there are 6 levels of pollution, labeled with different colors symbolizing the levels of concern, namely, good (green), moderate (34), unhealthy for sensitive groups (orange), red (unhealthy), purple (very unhealthy), maroon (hazardous). The levels of pollution are categorized according to the index values, which is computed from the equation as shown below. Table 5 present the classifications of AQI recommended by U.S. EPA.


$$\begin{array}{l} I_{P}\;=\frac{I_{Hi}-I_{Low}}{{BP}_{Hi}-{BP}_{Low}}\left(C_{p}-{BP}_{Low}\;\right)+I_{Low} \end{array}$$

I_p_ = Index of pollutant P
C_p_ = Concentration of pollutant P
I_Hi_ = AQI corresponding to Index of BP_Hi_
I_Low_ = AQI corresponding to Index of BP_Low_
BP_Hi_ = Breakpoint that is higher than or equal to C_P_
BP_Low_ = Breakpoint that is lesser than or equal to C_P_

**Table 5:** Recommendation of Air Quality Index (AQI) classification by U.S. EPA (32).

**AQI color**	**Index value**	**Pollution level of concern**	**AQI description**
Green	0–50	Good	Air pollution and satisfactory air quality pose little or no harm.
Yellow	51–100	Moderate	Air quality is adequate. Some people, however, may be at danger, particularly those who are highly sensitive to air pollution.
Orange	101–150	Unhealthy for sensitivity groups	Members of the sensitive group may suffer health consequences. Less liked to have an impact on the broader populace.
Red	151–200	Unhealthy	Some members of the general population may suffer from health consequences, while members of sensitive groups may suffer from more significant health problems.
Purple	201–300	Very unhealthy	Health warning: Everyone is at elevated risk of adverse health impacts.
Maroon	≥301	Hazardous	Everyone is most likely to be impacted by emergency situations, according to a health warning.

To determine the pollution level of the air, EPA has established National Ambient Air Quality Standards (NAAQS) based on the Clean Air Act. The NAAQS is developed for the primary pollutant, which is hazardous to public health and the environment, namely carbon monoxide, lead, nitrogen dioxide, ozone, PM particulate, and sulfur dioxide. Table 6 present the NAAQS table and illustrate the recommended threshold of the concentration of the pollutant in assessing the air quality.

**Table 6:** Recommendation of pollutant concentrations by National Ambient Air Quality Standards (NAAQS) by U.S. EPA (35).

**Pollutant**		**Average time of exposure**	**Level of exposure**
Carbon Monoxide (CO)		8 h	9 ppm^*^
		1 h	35 ppm^*^
Lead		3 months average	0.15 ug/m^3^^***^
Nitrogen Dioxide (NO_2_)		1 h	100 ppb^**^
		1 year	53 ppb^**^
Ozone (O_3_)		8 h	0.070 ppm^*^
Particulate matters / particles pollutions	PM_2.5_	1 year	12.0 ug/m^3^^***^
		1 year	15.0 ug/m^3^^***^
		24 h	35.0 ug/m^3^^***^
	PM_10_	24 h	150.0 ug/m^3^^***^

* *ppm = parts per million by volume*.

** *ppb = part per billion by volume*.

*** *ug/m^3^ = micrograms per cubic meter or air*.

To standardize the quality standard, WHO (1) had established the air quality guidelines in 2005 and published them in 2006, namely Air quality guidelines – global update 2005. The purposes of the guidelines are specific to provide evidence-based recommendations in form of air quality guidelines (AQG) levels, as well as an indication of the shape of concentration-response function (CRF) in connection to adverse health outcomes for pollutants such as particulate matters (PM). Nitrogen dioxide (NO_2_), ozone (O_3_), sulfur dioxide (SO_2_), and carbon monoxide (CO). It is useful for both long- and short-term pollutant exposure. The established guidelines are important to meet the air quality guidelines by providing interim targets, which to direct the reduce-effort activities. In addition, the guidelines play an important role in providing good practice statements in particular types of particulate matter management where there is insufficiency of evidence in deriving quantitative air quality guidelines threshold but emphasizes the health significance. According to the guidelines, recommendations such as air quality guideline (AQG) and the interim targets are given for the primary pollutants mentioned above as illustrated in Table 7.

**Table 7:** Summary of Air Quality Guidelines (AQG) levels and interim targets recommendations by WHO (36).

**Pollutant**	**Average time**	**Interim Target 1 (IT-1) μg/m^**3**^**	**Interim Target 2 (IT-2) μg/m^**3**^**	**Interim Target 3 (IT-3) μg/m^**3**^**	**Interim Target 4 (IT-4) μg/m^**3**^**	**Air Quality Guidelines Levels μg/m^**3**^**
PM_2.5_	Annual	35	25	15	10	5
	24-h	75	50	37.5	25	15
PM_10_	Annual	70	50	30	20	15
	24-h	150	100	75	50	45
O_3_	Peak Season	100	70	-	-	60
	8-h	160	120	-	-	100
NO_2_	Annual	40	30	20	-	10
	24-h	120	50	-	-	25
SO_2_	24-h	125	50	-	-	40
CO^*^	24-h	7	-	-	-	4

* *In mg/m^3^*.

However, in Malaysia, Department of Environment, Ministry of Environment and Water has established its own ambient air quality measurement which is closely referred to the PSI prepared by U.S. EPA. The air quality measurement in Malaysia is presented as Air Pollutant Index (API). The pollutants were monitored at varied average times in accordance with the standards given by WHO, based on the requirements of the Malaysian Ambient Air Quality Standard (MAAQS) in terms of human health consequences (37). In Malaysia, API are categorized into 5 categories as presented in Table 8.

**Table 8:** Air Pollution Index classification recommended by Department of Environment (DoE), Malaysia (37).

**Air Pollution Index (API)**	**API status**	**Color**	**API description**
0–50	Good	Blue	There is little pollution, and these are no harmful health effects.
51–100	Moderate	Green	It has no harmful effects on health.
101–200	Unhealthy	Yellow	Sensitive folks should avoid. Health conditions for the elderly, pregnant women, children, and persons with heart and lung issues deteriorate.
201–300	Very Unhealthy	Orange	Unhealthy for the public. Worsening health and a reduced tolerance for physical activity might lead to lungs and heart issues.
>301	Hazardous	Red	Emergency

To compute the API, the New Ambient Air Quality Standard was developed in accordance to standardize the air pollutant concentration threshold. The pollutants involved are particulate matters (PM_10_ and PM_2.5_), ozone (O_3_), sulfur dioxide (SO_2_), carbon monoxide (CO), and nitrogen dioxide (NO_2_). Table 9 shows the air pollutants concentration levels adopted by the New Ambient Air Quality Standard.

**Table 9:** Ambient air quality standard in Malaysia by Department of Environment (DoE), Malaysia (38).

**Pollutant**	**Averaging time**	**Ambient air quality standard**
PM_10_	Annually	40 μg/m^3^
	24 h	100 μg/m^3^
PM_2.5_	Annually	15 μg/m^3^
	24 h	35 μg/m^3^
SO_2_	1 h	250 μg/m^3^
	24 h	80 μg/m^3^
NO_2_	1 h	280 μg/m^3^
	24 h	70 μg/m^3^
O_3_	1 h	180 μg/m^3^
	24 h	100 μg/m^3^
CO	1 h	30 mg/m^3^
	24 h	10 mg/m^3^

#### Particulate Matters

Particulate matters (PM), which are common airborne particles, is a complicated mixture of solids and aerosol made up of minute liquid droplets, solid fragments and solid cores coated in liquid (39). PM has been categorized into 3 categories based on the particle sizes, namely coarse particulate matter (PM_10_), fine particulate matter (PM_2.5_), and ultrafine particulate matter (PM_0.1_). The difference between PM_10_ and PM_2.5_ is the aerodynamic diameter where PM_10_ has a diameter of 10 μm while PM_2.5_ has a diameter of 2.5 μm. Diameter in ultrafine particles is the smallest, which is smaller than 0.1 μm.

PM_2.5_ particles are often composed of substances such as sulfate (SO_2_). Nitrate (NO), ammonium (NH^+^), organic compounds, and metal such as lead (Pb), Cadmium (Cd), vanadium (V), nickel (Ni), Copper (Cu), and Zinc (Zn), and hydrocarbons (40). The sources of the fine particles, PM_2.5_ normally can be found in the combustion of coal, oil, and gasoline. It is also produced from the transformation of NO_x_ and SO_2_. High temperature processes such as smelters and steel mills tend to produce the PM_2_._5_. Based on (41), the presence of PM_2.5_ in the air or the lifetime of PM_2.5_ can be last from days to weeks, meanwhile, PM_10_ only lasts for minutes to hours. Fine particles (PM_2.5_) have a travel distance of 100 to 1000 kilometers, while the travel distance is limited to only 1 to 10 km (42). Coarse particles (PM_10_) composed of dust such as soil dust, resuspended dust, street dust and oil fly ash. Metal oxides such as silicon (Si), aluminum (Al), Titanium (Ti), calcium carbonate (CaCO_3_) etc. and substances such as pollen and mold spores are categorized under coarse particles. Particles which are having course size often produce from soil track, roads and streets, suspension from soils and industrial dusts. Coal and oil combustion also play an important role in the coarse particle formation (42). High concentration of particulate matters distribution often happens in the area or nearby sources such as industrial area, agricultural activities, and fuel combustions.

High levels of PM pollution are well-known in causing adverse health in humans, no matter whether under short-term or long-term exposures. The most common and major health effects reported are cardiovascular and respiratory morbidity and mortality. Other effects such as adverse fatal development and infancy, induction or exacerbation of diabetes mellitus and cancer have been linked to the consequences of PM exposure (43–48). The impact of PM exposure to cardiovascular and respiratory systems should not be underestimated. In respiratory system, pollution has become the key contributor to the Global Burden of Diseases (GBD) (49, 50). Based on the GBD 2015, PM, specifically PM_2.5_,was the fifth leading cause of death, contributed to 4.2 million of death, that represent global deaths of 7.6%, 10.31 million were facing disability-adjusted life-years (DALYs), which is about 4.62% of global DALYs rate (51). According to Cohen, Brauer (51), various studies have been done in proving the effect PM_2.5_ exposure that is closely associated with the variety of respiratory diseases. There has been positively increment in the tendency of respiratory infections, outpatient visits frequency, emergency visit and hospitalization frequency for respiratory infection.

Inhalation of particulate matters increases the exposure and adverse health effect in respiratory system. Fine particles (PM_2.5_) possess the greatest risk to human health where the particle size fine enough to penetrate deep into human respiratory tracts and lungs, as well as the bloodstream while PM_10_ has a lower risk in causing human health effect due to its coarse size. Although PM_10_ can cause irritation to eyes, nose and throat, due to the bigger size particle, it is unable to penetrate into bloodstream and lungs (52). Based on (53), particles size between 5 μm to 10 μm tends to be deposited in tracheobronchial tree whereas particles with diameter of smaller than 5 μm is more susceptible to enter the respiratory bronchioles and alveoli, where gaseous exchange takes place. Fine particles with diameter smaller than 1 μm has a similar characteristic and as gas molecules, they able to diffuse into the bloodstream, subsequently translocate into cell tissue and/or circulatory system (54). According to (40), studies concluded that PM particles also tend to trigger airway injury and inflammation which increased the production of reactive oxygen species (ROS) in vivo, consequently damage in cellular and tissue. Besides, PM particles also contributed to high impact on lungs and cardiovascular system diseases by inducing the pro-inflammatory cytokinesis production. These genotoxic effects in human lung cells, have majorly caused causing oxidative stress (40).

Many experimental studies have been and successfully proven that the exposure of PM_2.5_ potentially makes the body more vulnerable to pathogens by weakening the respiratory host defense. Impairment of respiratory host defense included defective airway epithelial host defense functions, alterations in respiratory microecology and insufficiency and dysfunction of immune cells (55). A healthy and normal airway epithelial is free from pathogens, protected by a barrier function of epithelium, namely mucociliary clearance. (55) and (56), claims that fine particles (PM_2.5_) exposure impairs the bronchial mucociliary system, which reduces bacterial clearance. The exposure of PM_2.5_ has disrupted the defense function of the mucociliary system, as well as the secretion of antimicrobial peptides, hence, increase the vulnerability of the body to pathogens.

In addition, studies found that upper airway in human consisted of bacterial flora, which is a crucial natural immune defense mechanism. The bacterial flora established a biological barrier against foreign materials and harmful germs by a space-occupying effect, nutritional competition and release of bacteriostatic or bactericidal chemicals (57–59). Moreover, PM_2.5_ contributed to insufficiency and dysfunction of immune cells, causing adverse health impact in humans. PM_2.5_ exposure has reduced the phagocytic phagocytosis and increased the chance of pneumonia, by getting *S.aureus* infection (48). Besides, PM_2.5_ reduced the immune system by declining the phagocytic capacity of macrophages, diminishing the production of antimicrobial oxidants in response to *L. monocytogenes* in macrophages. This is crucial as low production of antimicrobial oxidants increased the chance of respiratory infections. *L. monocytogenes* infection, bacterial infections often lead to meningitis, as well as spontaneous abortions in pregnant women. Not forgetting the impact of PM_2.5_ on metabolic activation, it is frequently found in laminar organelles. PM_2.5_ will have a variety of harmful consequences on the targeted cells. This can be occurred due to the metabolically activation of organic chemicals in PM_2.5_ by xenobiotic-metabolism enzyme system. The aryl hydrocarbon receptor (AhR) is activated by the release of organic molecules (VOCs and PAHs) from PM_2.5_. Subsequently, enhanced the AhR-regulated gene expression which forms the xenobiotic-metabolism enzyme system (60).

Furthermore, exposure to PM particles induce cardiovascular diseases such as ischemic heart disease, heart failure, as well as cerebrovascular diseases (61). Studies proved that the risk of myocardial infraction, fatal and non-fatal ischemic heart diseases and heart failure incident are associated with the ambient exposure no matter in acute or chronic exposure (31, 62–67). According to WHO (1), 3.7 millions of death are caused by the outdoor ambient pollution, 80% of deaths are resulted from heart diseases and stroke. As mentioned earlier, fine particles and ultrafine particles are small in sizes, and due to their chemical composition and charge, the particles are able penetrate through the bloodstream, and move to the other parts of the body.

A low concentration of PM exposure in the blood stream has a high possibility in cumulating toxicity (68). According to (69), PM exposure has both direct and indirect actions onto the cardiovascular system. These include the deposition of the particles onto the vascular endothelium, an inner layer of blood vessels that contact the blood, could result in the local oxidative stress and inflammation. Subsequently, resulting the instability of atherosclerotic plaque and thrombus formation (70). In addition, according to (71), PM causes elevation of ejection fraction and premature ventricular beats. These events often lead to adverse impact onto cardiac especially patients with coronary heart disease by increasing the unhealthy cardiac oxygen needs and exacerbate the ischemia condition. Besides, the indirect effect of PM is also crucial in contributing burden to cardiovascular system. The indirect event often caused by the inflammation in lungs, lead to atherosclerosis progression and blood coagulability and endothelial dysfunction, finally exacerbate myocardial ischemia (69). In addition, exposure of PM is hypothesized to activated the autonomic nervous system (ANS), causing autonomic balance to be disrupted and sympathetic tone to be favored (72, 73). (73) claimed that an elevated risk of cardiovascular disease is linked to the overactive sympathetic tone, by inducing prohypertensive vasoconstriction and proclivity for arrhythmias. In investigating the impact of PM exposure on the cardiac, the modulation of microRNAs (miRNAs) has become one of the risk factors to be investigated on. This is especially critical when involved in systemic inflammation, endothelial dysfunction and atherosclerosis (74, 75).

Often, PM pollution is concerned among the public and researchers due to its adverse impact on human health and climate. According to WHO suggestion, the concentration of PM_2.5_ should not exceed 10 μm/m^3^ annually and 25 μm/m^3^ daily to ensure the cleanliness of the air while the concentration of PM_10_ should be controlled within 20 μm/m^3^ annually and 50 μm/m^3^ daily. This is to ensure the concentration of the particles in the atmosphere is within the healthy level and has a low health impact on humans. Due to the various sources of PM particles, it is not an easy task to determine the peak concentration, or the trend of PM particles released in the atmosphere daily and annually. To monitor the concentration of PM particles, Air Quality Index (AQI) plays an important role in determining the air quality. AQI in Malaysia is adopted from the guidelines provided by U.S. EPA. To assess the air quality, the PM concentration often referred to the AQI as a baseline to determine the concentration level and its impact on humans. Therefore, it is important to establish a system in forecasting the PM concentration, concurrently, predict the peak concentration of PM particle daily and annually according to the AQI.

#### Carbon Monoxide

Carbon monoxide (CO), a colorless and odorless gas that is produced from incomplete combustion (76). Carbon monoxide (CO) is often produced during combustion with low oxygen level or low mixing, which often happens in motor vehicles, power stations, waste incinerators, domestic gas boilers and cookers. Although CO is not poisonous, it possesses an adverse temporary effect on the human respiratory system by inhibiting the oxygen uptake in hemoglobin. This is due to the nature of CO, which has a far higher affinity to hemoglobin than oxygen, which leads to severe poisoning in case of long-term CO exposure (77). Ones inhaling excessive CO will experience symptoms such as dizziness, headache and nausea, while experience disorientation, unconsciousness and death (78).

#### Sulfur Dioxide

Sulfur dioxide (SO_2_), a colorless gas in physical characteristic, alongside a choking and suffocating odor. SO_2_ is primarily produced by human activities for instance, combustion of coal and oil, often happening in power plants, or from copper smelting. Meanwhile, it can also be produced or released naturally into the air by volcanic eruptions (79). According to WHO, SO_2_ shows adverse effect on sensitive cohort such as asthmatics patients with respiratory diseases (36). This is due to its ability to penetrate the body, mainly through the respiratory system. Exposure to SO_2_ often contributed to daily death number and hospital admissions for respiratory or cardiovascular causes such as asthma and chronic obstructive pulmonary disease (COPD). In long-term exposure to SO_2_, studies proved that SO_2_ plays an important role in the frequency and possibility of wheeze and symptoms such as cough and phlegm (80–82). Chronic effects of SO_2_ exposure included decrement in lung function or changes in the lung function. SO_2_ tends to cause inflammation on the respiratory tract and raises the risk of infection. Moreover, excessive SO_2_ exposure could induce coughing and mucus flow, aggravates asthma and chronic bronchitis.

#### Nitrogen Oxide

Nitrogen oxides (NO_x_) is a big umbrella that indicated a group pollutants gases produced by the reaction between nitrogen (N_2_) and oxygen (O_2_) (83). NO_x_ is a colorless and odorless gas which is also formed by fuel combustion at high temperature. NO_x_ is formed when the “fixation” of nitrogen in the dilution air into NO_x_ under a high temperature (84). The pollutant gases under the umbrella of NO_x_ are NO, NO_2_, N_2_O, NO_3_, N_2_O_3_, N_2_O_4_, and N_2_O_5_(85). The main sources of NO_x_ are mobile and stationary sources such as motor vehicles or transportation fuel combustion, and power or heating generation. Often, the major or predominant nitrogen oxides involved in the air pollution are nitrogen dioxide (NO_2_) and nitric oxide (NO) (86). NO_2_, which is visible in reddish brown, along with particles in the air. In urban areas, when the pollution of NO_2_ occurs, a layer of reddish-brown color can be seen over the air. Meanwhile, nitric oxide is formed from the oxidation of nitrogen dioxide. Although the gases are colorless and odorless, it causes an adverse and harmful effect to the health when penetrates the body through inhalation. Excessive inhalation of the NOx will irritate the respiratory system, and induce diseases such as coughing, wheezing, dyspnea, bronchospasm, and pulmonary edema (77). According to U.S. EPA (87), NO_2_ plays an important role in increasing inflammation of airways and asthma attacks, especially in children, reducing lung function, as well as increasing the possibility of emergency and hospitals admission. Long-term exposure of NO_x_ tends to impact health by causing chronic lung diseases, increase the chance of impairing the smelling sense in human. It also causes irritation symptoms to eyes, throat and nose. Based on (88, 89), NO_2_ concentration often shows the highest reading in urban regions, especially in urban areas where the roadways or traffic are busy.

#### Ozone

Ozone (O_3_), a gas molecule that is made up of three oxygen atoms in the presence of a third-body molecule capable of absorbing the reaction's heat. The stratosphere, a layer high in the upper atmosphere which protects humans from the UV radiation produced by the sun. However, when the ozone air pollution happens in the troposphere, which is at the ground level, it is harmful to humans when inhaling, caused major health concerns (90). The upper respiratory tract absorbs most of the ozone after inhalation, which is carried into the intrathoracic airways. Often, oral inhalation also allows ozone absorption into the body, a relatively lower rate. However, higher penetration of ozone into the lung is possible to occur when carrying out an aggressive activity (91). (92) and (77) claims ozone-induced toxic consequences have been documented in metropolitan areas all over the world, raising issues in biochemical, morphologic and functional and immunological aspects. According to (91), ozone causes adverse health effects by dissolving in a thin layer of epithelial lining fluid in the lower respiratory tract after inhalation. Due to its characteristic of low solubility in water, ozone is unable to be removed effectively from the body by upper respiratory tract. It can only be removed as secondary oxidation products that caused oxidative stress, contributed to cellular injury and altered cell signaling in respiratory tract, inflammation as well as chemokines and cytokines, vascular endothelial adhesion molecules. Besides, the ozone contributed to acute and chronic health effect such as mortality, pulmonary system effect caused by ozone exposure relative to respiratory health, cardiovascular diseases such as vascular oxidative stress, elevated heart rate and diastolic pressure, inflammation, and decreased heart rate (93, 94). Meanwhile, in chronic health effects, asthma, life expectancy reduction and lung function effects as well as atherosclerosis are often linked to the consequences of long-term exposure of ozone (O_3_) (91).

#### Heavy Metals

In air pollution, heavy metals play an important role in causing irreversible effects on human health. Not only to human health, but heavy metal contamination is also threatening the environment and ecosystem. There are various heavy metals such as lead (Pb), arsenic (As), copper (Cu), mercury (Hg), Cadmium (Cd), Vanadium (V), Titanium (Ti) and etc. Due to the emergence of anthropogenic activity and increase of the use of heavy metals, mining, smelting, foundries as well as leaching of metals occurred. These activities are important in contributing to heavy metal pollutions (95). Hence, it has impacted the terrestrial and aquatic. Exposure to heavy metals is often related to adverse health effects such as DNA damage, causing carcinogenic effects, cell or cell membrane damage, cellular function reduction as well as neurotoxicity where the nervous system is damaged. Among the heavy metals that contributed majorly to pollution are lead (Pb), arsenic (As), copper (Cu), mercury (Hg), Cadmium (Cd), and nickel (Ni). Lead (Pb) exposure often occurs through inhalation of dust particles or aerosols contaminated with Pb, as well as through consumption of polluted sources of food (96). According to (97), high exposure or poisoning of Pb has a likelihood of damaging the kid ney, liver, heart, brain, skeleton and nervous system. It also causes auditory impairment, gastrointestinal damage and cognitive skills and health. Cancers and diseases such as Alzheimer's have a higher possible in the exposure of Pb (98). Meanwhile in Cadmium (Cd), according to (96), studies show that excessive exposure to Cd can damage respiratory, cardiovascular, renal, skeletal system and cancer development. Workers from metal industries are the major cohort to expose to Cd (99). On the other hand, exposure to mercury can be caused by consumption of agricultural products and dental care-amalgams (100). The effect of ingestion of mercury includes gastrointestinal toxicity, neurotoxicity, and nephrotoxicity as the mercury exposure are being aggregated in kidneys liver and neurological tissues (101). According to WHO, arsenic, a highly toxic metal which often exposed to human by eating, drinking, and preparation of food and irrigating crops using contaminated water as well as industrial processes (102). Chronic health effects due to long-term exposure of arsenic included skin lesions, patches on limbs (palms and soles), cancers such as skin cancer, lung cancer and bladder cancer. Besides, long-term exposure of arsenic also causes adverse pregnancy outcomes such as infant mortality, development defects and cognitive health. It could also cause irreversible effects such as pulmonary disease, cardiovascular disease, and myocardial infraction. And worst, it could cause mortality (103, 104).

#### Other Pollutants (VOCs and PAHs)

Pollutants such as volatile organic compounds (VOCs) and polycyclic aromatic hydrocarbons (PAHs) have toxicology potential which is harmful to human health. Volatile organic compounds are known as a crucial pollutant that existed in variety of industrial fields (105). VOCs presented in the emission of fossil fuels and motor vehicles in the ambient air (106). However, it is more common to find VOCs in indoor pollution, which is produced from paints, photocopy machines and air fresheners and disinfectant. Compounds such as toluene, benzene, and xylene are under the big umbrella of VOCs, which have been linked to the development of cancers in humans (77). Exposure to VOCs contributed to both short term and long-term health effects such as hazardous and toxic reactions, irritation of the eyes, nose, throat and mucosal membranes (107). Besides, exposure to high levels of VOCs can lead to cancers and central nervous system damage as chronic exposure effect.

Polycyclic aromatic hydrocarbons (PAHs) are a group of organic compounds that are composed of two or more fused aromatic rings. Compound such as naphthalene, benzo[a]pyrene, pyrene, fluorene, acenaphthylene and etc are under the umbrella of PAHs and these compounds are labeled as high priority pollutants (108). PAHs often produced by incomplete combustion of fuels and organic materials such as coal, petrol, oil and wood. It can also be found in the food, water, soil and plant (106, 109). Due to its presence in the food and water, especially in barbecue, it could cause cancer due to the carcinogens present in the food. PAHs can also be found in indoor pollution sources such as tobacco smoke and wood stove (106). According to (77) PAH compounds are recognized as an crucial risk factor for lung cancer which they is toxic and contains mutagenic and carcinogenic substances. Exposure of PAHs can occur through ingestion, inhalation, and dermal contacts (110). The most significant exposure of PAHs is through the inhalation of PAHs into the human body. Exposure of PAHs contributed to adverse health effects such as the risk of lung cancer (111). Acute excessive exposure to PAHs possesses symptoms such as irritation of eyes, nausea, vomiting and diarrhea (15). Chronic health effects such as immunity declined, organ damage, cataracts, respiratory issues such as breathing problems and asthma-like symptoms, as well as lung function abnormalities. Ones may also experience inflammation on skin after repeated contact (110).

## Discussion

Therefore, realizing the impact of air pollution on health, we propose a new framework of an integrated environmental and health impact assessment system through the development of federated learning network architecture. This is important to assess the potential risk contributed by air pollutants to human health. The output of the impact assessment is critical in healthcare management and monitoring, and to enhance the quality of healthcare services. In this section, we will also highlight our proposed contributions to the techniques applying in the integrated system, namely federated learning.

### Integrated Environmental and Health Impact Assessment

Air pollution is among the primary contributors to climate change, and currently it is the most significant environmental challenge (112). Air pollution contributed to irreversible effects to human health. As presented in the previous section, urban air pollution particularly has become a global threat to human welfare and health. This situation has been affecting more than half of the world's population and it is growing and will continue to grow reaching 70% by the year 2050 (113–115). Hence, it is crucial to establish an artificial intelligence (AI) based integrated environmental and health decision system to assess the risk of environmental pollution to the health particularly on the air pollution. AI has been widely used in a variety of fields in improving effectiveness of automation and analyze data at a much faster rate than humans. From (116), AI has extended its application to environmental management problems, including in modeling water quality, fish stock prediction and other environmental engineering applications.

In the twenty-first century, AI is widely applied in various fields. AI and its subsets machine learning and deep learning has been the backbone of decision support system, monitoring and predictive tools, often involved in engineering, medical, analytics etc. As from the review performed, it is clearly presented that AI has been extensively used in monitoring the environmental issues associated with the health impacts. The AI techniques helped in assessing the relationships between environmental pollution and health impacts, subsequently study the trend of the associations without human interventions. The AI techniques that commonly applied are machine learning such as random forest, decision tree, Naïve Bayes, SVM, regression models etc, while the deep learning techniques such as ANN, long short-term memory (LSTM), DNN etc. In addition, assessment of the association between pollution and health impacts has been performed using techniques such as quasi- poison generalized linear regression, generalized additive mixed model (GAMM), geographic weighted poison regression etc. These are the common models used in the previous studies.

However, there are limitations from previous studies. According to the performed review as shown in Table 3, where the techniques used are almost similar. Generally, techniques such as decision tree, random forest and regression models are the most common techniques used by scientists in prediction of air pollution and the association between its impact to human health. Although these are well-known and powerful techniques, their accuracy prediction is depended on the data being processed. Besides, the major limitations such as insufficient data on pollutant information and insufficient monitoring stations also present as the research gap of the studies. Moreover, insufficient variables also directed as the study limitations. In addition, there is lacking studies and evidence in air pollution predictive system in Malaysia. This review paper aims to propose an AI integrated environmental and health impact assessment.

The integrated environmental and health impact assessment is an integrated system which acts as a means for monitoring the environmental pollution and its impact to human health, concurrently, assesses the health impact on human after the pollution. This is to provide and support a more comprehensive decision-making. This system has handful approaches in establishing the integrated system, which are system dynamics, Bayesian networks, coupled component models, agent-based models and expert systems (117). In this study, the construction of the integrated system is developed according to a structured framework (118). A good, structured framework allows simultaneous monitoring and interpretation, and provides a systematic way in discovering linkages or correlations between the environment and human health (119). According to the (119), a robust framework should have 4 elements, namely i) conceptual clarity and scope, ii) flexibility, iii) balance, and iv) usability. INTRASE project completed by author (120) developed an integrated environmental and health impact assessment framework which assess the health-related problem derived from the impact of policies related to environment and health, as well as other factors and interventions that affecting the environment (118). The framework considers the complexities, interdependencies and uncertainties in reality (119, 121). The framework comprised of 4 stages namely, (i) issue framing to define the problem and aim for the assessment, concentrates, restricts the scope of evaluation and management alternative. (50) Design stage with the goal to transform conceptual model developed during issue framing into a comprehensive evaluation process. (iii) Execution stage which is the heart of the evaluation procedure. (iv) Appraisal which the outcome synthesis and interpretation (118).

**Figure 2:**
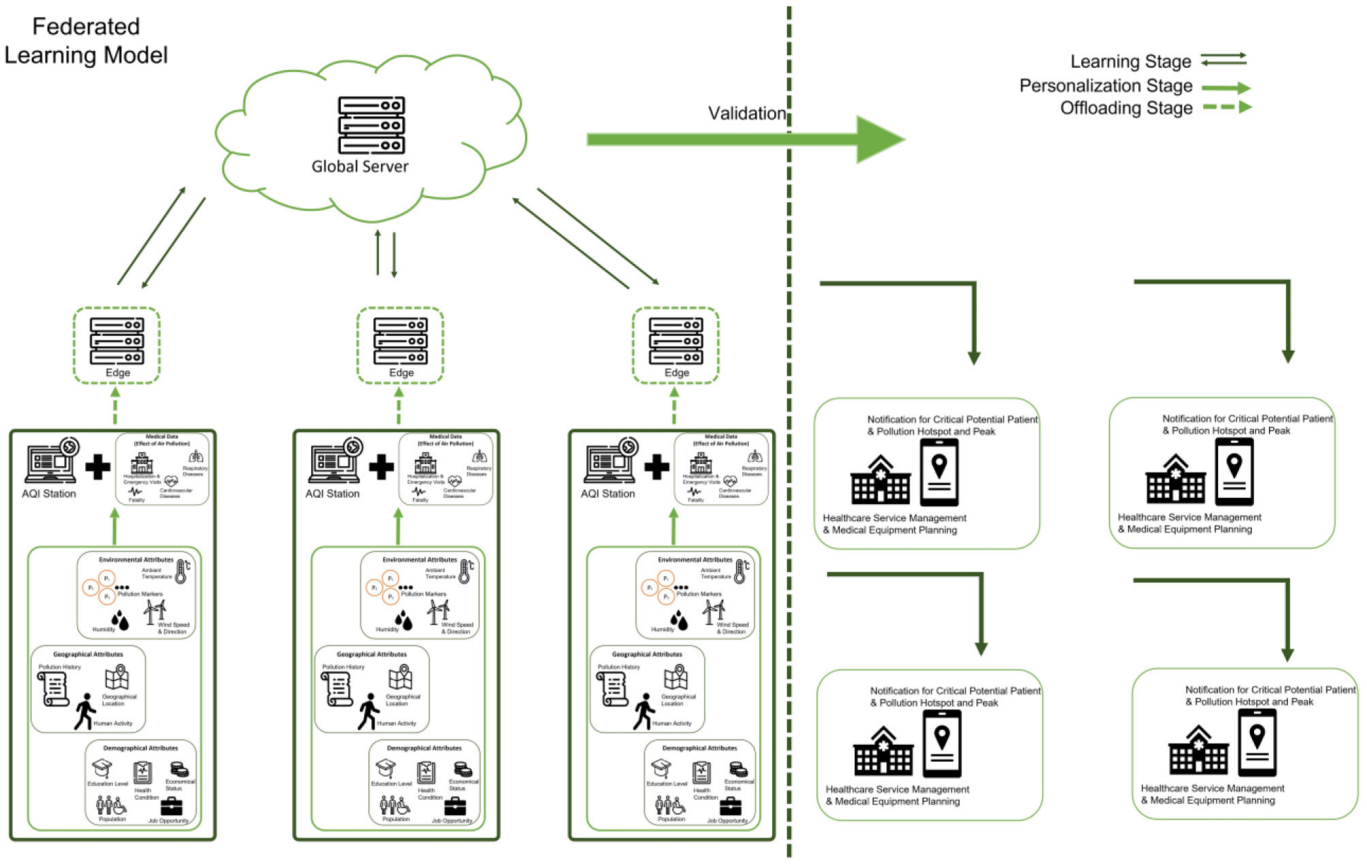
Framework of integrated environmental and health impact assessment.

In this study, the proposed conceptual framework of the integrated assessment system is constructed as Figure 2. The integrated system is feed with input parameters, related to data such as meteorological data, wind speed, wind direction, weather, humidity, atmospheric pollutant data of particulate matter, ozone, carbon monoxide, sulfur dioxide, and nitrogen dioxide and volatile organic compounds (VOCs); data of health impacts of air pollution; geographical and socio-economic activities. Missing and incomplete dataset can be solved based on the predictive models using the parameters retrospective data. The data input into the models is significant for the correlational studies between air pollutant and health impacts, based on socio-economics activities. The significant outputs of the model will serve as the input variables of the air quality index predictive models. The AQI predictive models are crucial in generating the measurement of air quality, monitoring the trend of the pollution. The AQI model is the core model to identify the pollution threshold concentration of the ambient pollutant. It is critical for air quality standard monitoring and policy making. In addition, the impact assessment environmental pollution and health can be carried out with the output information of AQI predictive model. The impact assessment is crucial in determining the potential impact of pollution to the human health, hence, aids in hospital and health care services planning and management, which is the main research gap in the study. The planning and management of hospital and healthcare services is critical in early preparation of services to accommodate the needs and ensure the sustainability of public health.

### Federated Learning: Towards Integrated AQI Monitoring Solution

The proposed integrated system will be developed using federated learning (FL) techniques as the foundation of the predictive models. Federated learning is an evolving machine learning approach aimed at addressing the problem of data island while protecting data privacy (122), proposed by Google (123–125). Federated learning is named on its characteristic which the learning task is performed by multiple clients, or an informal network of participating devices for instance mobile devices (126). It is coordinated by a central server for decentralized machine learning settings. According to author (122), FL closely associated with distributed learning. It connects numerous computers in various places over a communication network under the management of central server, so that each computer completes different sections of the same operation. This proved that distributed processing is concerned with the n accelerating processing stage whereas FL is primarily concerned with developing a collaborative approach that does not compromise privacy. With its novel architecture, FL permits multiple participants to build a machine learning model jointly while keeping their private training data private. FL is thought of as privacy-preserving, decentralized collaborative machine learning. It has also been seen as an operating system for edge computing since it offers a learning protocol for coordination and security (127).

Federated learning, as an innovative modeling that can train a single model on data from multiple sources without jeopardizing the privacy and security of those data, has promised its application in industries such as sales, finance, healthcare etc. Due to intellectual property rights, privacy protection and data security, these data cannot be directly aggregated for training machine learning models (127). Looking at this study, to address the privacy concern in AQI prediction and impact assessment, FL is implemented as the environmental data and medical data are very sensitive and private. This is critical when the environmental agencies and medical services collaborate in monitoring and defeating the issues of pollution and its impact on health. Several research have implemented FL in their studies. For example, authors (128–130) have implemented FL in monitoring air quality and forecasting. FL is implemented in these studies as FL contributed to protect the data when monitoring without sharing the raw data, for instance the data images from unmanned aerial vehicles (UAV). Besides, the reason of implementing FL includes its flexibility, which FL learning can be performed when the devices are in charging mode, on or not on the internet or WiFi connection [135]. Besides, FL also enables real time prediction. There is no need to be concerned about any time lag while sending information to and from the server. Also, FL only requires a minimal infrastructure, it does not require intensive hardware. These advantages are promising to be applied in air quality monitoring contact especially in the developing countries as Malaysia. Since the air quality monitoring stations are scattered throughout the country, the feasibility of processing air quality attributes using decentralized collaborative machine learning in FL will enable faster diagnosis and thus rapid environmental decision making can be made. Since the FL does not require intensive hardware, the FL architecture can be easily embedded with the existing sensor networks at air quality monitoring stations.

Overall, FL has various promising potential to be utilized in predictive models. Although there is no research proven the feasibility of implementation of FL in the construction of integrated environmental and health impact assessment, there are studies proven that federated learning is famous particularly in data security as discussed above. The aim of this paper is to propose the implementation of FL in the integrated impact assessment system as it involved environmental and medical data.

## Conclusion

Air pollution has become a major environmental issue around the globe. Poor air quality affects human health by exposing to the poisonous air pollutants such as particulate matters, carbon monoxide, ozone, Sulfur dioxide, heavy metals, VOCs etc. Both short-term and long-term exposures are significant in contributing to adverse health effects such as respiratory diseases, cardiorespiratory diseases, irritation to eyes, nose and throat and worst, it can cause cancers to human. Hence, it is important to monitor air quality as different pollutants possess different threshold concentration of impurities. Prediction of air pollutant can reduce the health impacts caused by air pollution, especially to the sensitive groups of publics by forecasting the potential pollution. Besides, the impact assessment is also crucial to understand the potential adverse health effect contributed by air pollution. With the aid of the assessment, hospital management is able to provide effective and efficient medical services to the patients. Therefore, an integrated system of environmental and health impact assessment is proposed. In this context, AI is expected to serve as a predictive model technique in air quality index and integrated environmental and health impact assessment. This is due to the capability and feasibility of AI to identify trends and patterns from big data, open previously unimagined avenues for addressing complicated environmental issues.

From the study above, research on air pollution and its impact on human health revealed a fundamental comprehension of the relationships and association between air pollution and health hazards to humans. The study also provided insight on the prediction of human health due to air pollution and air pollution prediction using machine learning techniques. The ultimate benefits gained from this study is the need of a highly capable integrated impact assessment system to understand the effect on the health caused by environmental issues, which is air pollution in this context. This is critical in medical services preparation and prioritizing the critical patients affected by the air pollution.

According to the reviews and findings, it is found that there are insufficient studies performed on the prediction of impact of health caused by air pollution in Malaysia. In addition, there is also lacking research on the prediction of potential hospitalization and emergency visits associated with the impact of air pollution in Malaysia, which is mainly concerned to accommodate the healthcare services that prioritize the potential patients affected by air pollution. Hence, it is essential and critical for us to propose the integrated environmental and health impact assessment to curb the current issue and fill in the research gap. Therefore, this study provides recommendations that will be useful in future research:

Feasibility of developing an integrated system on environmental and health impact assessment to monitor the trend and pattern of the relationships of the pollutant sources, markers and effects on health, as an effort to solve complex interaction and serve as a pre-requisite for AQI predictive model.

AQI prediction in monitoring pollution markers levels based on socio-economic activities and geographical area. AQI serves as an indicator of health quality monitoring referral in sensitive groups. It is also critical in providing guidelines and reference in standards making.

Imposing a framework on integrated environmental and health impact assessment systems based on the information from environmental, health, and AQI prediction. The capability of AI and FL in learning and predicting the big data. It is also critical in hospital monitoring in the context of prediction of early health care services preparation and hospital management, for effective medical services.

Considering evidence, the review provided a new insight on prediction of air pollution and its impact to health, by adopting AI and FL. Concurrently, an integrated environmental and health impact assessment development aids in reducing future threats to human health and lifted the healthcare service quality and management.

## Data Availability Statement

The original contributions presented in the study are included in the article/Supplementary Material, further inquiries can be directed to the corresponding authors.

## Author Contributions

EN and KH designed and developed the study protocol as well as major contribution to the article writing. MM and KL performed the identification, screening, eligibility, quality assessment, and information extraction of the articles. MA, SR, and HH checked all the synthesized data and approved the final version to be submitted for publication. All authors have substantially contributed to the article.

## Funding

This work was supported in part by the Ministry of Higher Education through the Malaysian Research University Network (MRUN) Young Researchers Grant Scheme (MY-RGS) entitled “Climate Change Mitigation: Artificial Intelligence-Based Integrated Environmental System for Mangrove Forest Conservation” under Grant MR001-2019, and in part by the Universiti Malaya Research University Grant (SATU) entitled “Climate-Smart-Mitigation and Adaptation: Integrated Climate Resilience Strategy for Tropical Marine Ecosystem” under Grant ST065-2021.

## Conflict of Interest

The authors declare that the research was conducted in the absence of any commercial or financial relationships that could be construed as a potential conflict of interest.

## Publisher's Note

All claims expressed in this article are solely those of the authors and do not necessarily represent those of their affiliated organizations, or those of the publisher, the editors and the reviewers. Any product that may be evaluated in this article, or claim that may be made by its manufacturer, is not guaranteed or endorsed by the publisher.
