# Supplementary material for: Towards Integrated Air Pollution Monitoring and Health Impact Assessment Using Federated Learning: A Systematic Review
Source: Front Public Health. 2022 May 19;10:851553. doi: 10.3389/fpubh.2022.851553 (PMC9160600; doi:10.3389/fpubh.2022.851553)
Supplement: Supplementary file 1 [file Data_Sheet_1.pdf]

Supplementary Material

1 Supplementary Figures and Tables

1.1 Supplementary Figures

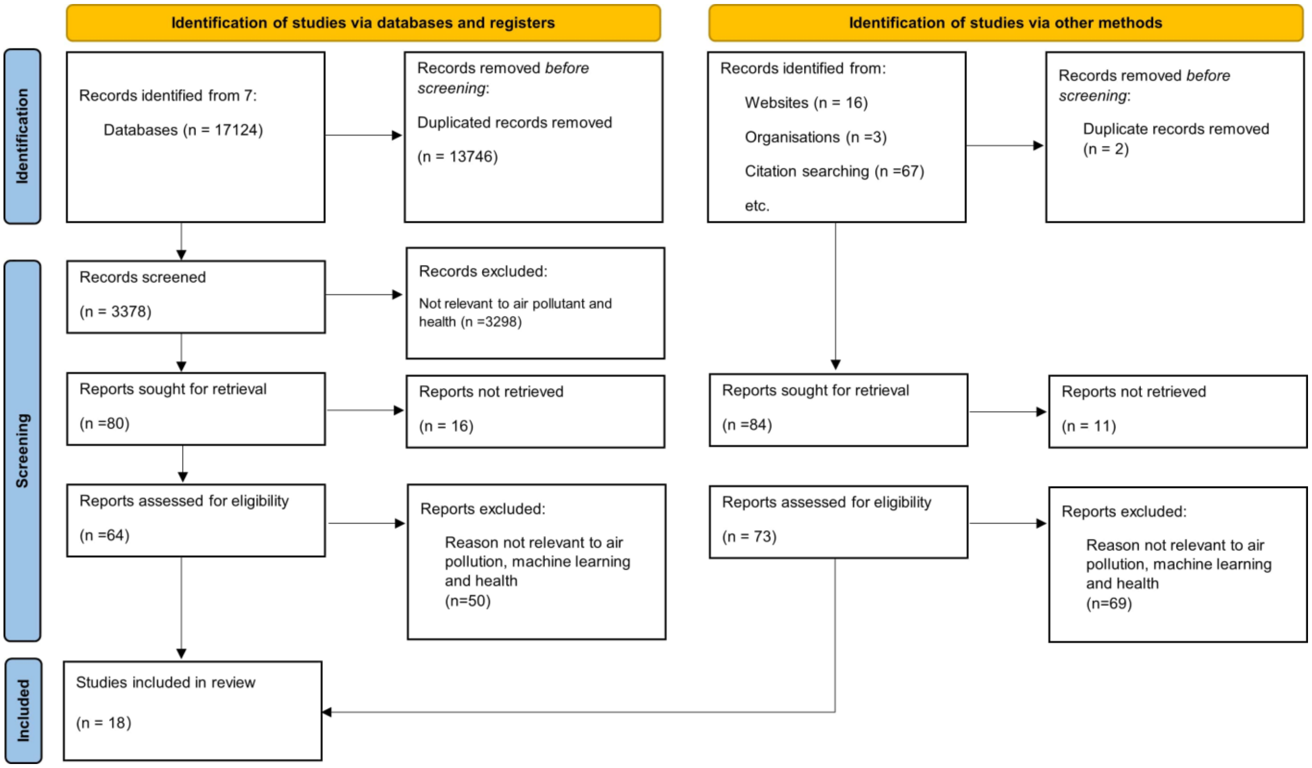

Figure 1 PRISMA flow chart of the review adopted from The PRISMA 2020 statement: an updated guideline for reporting systematic reviews (1).

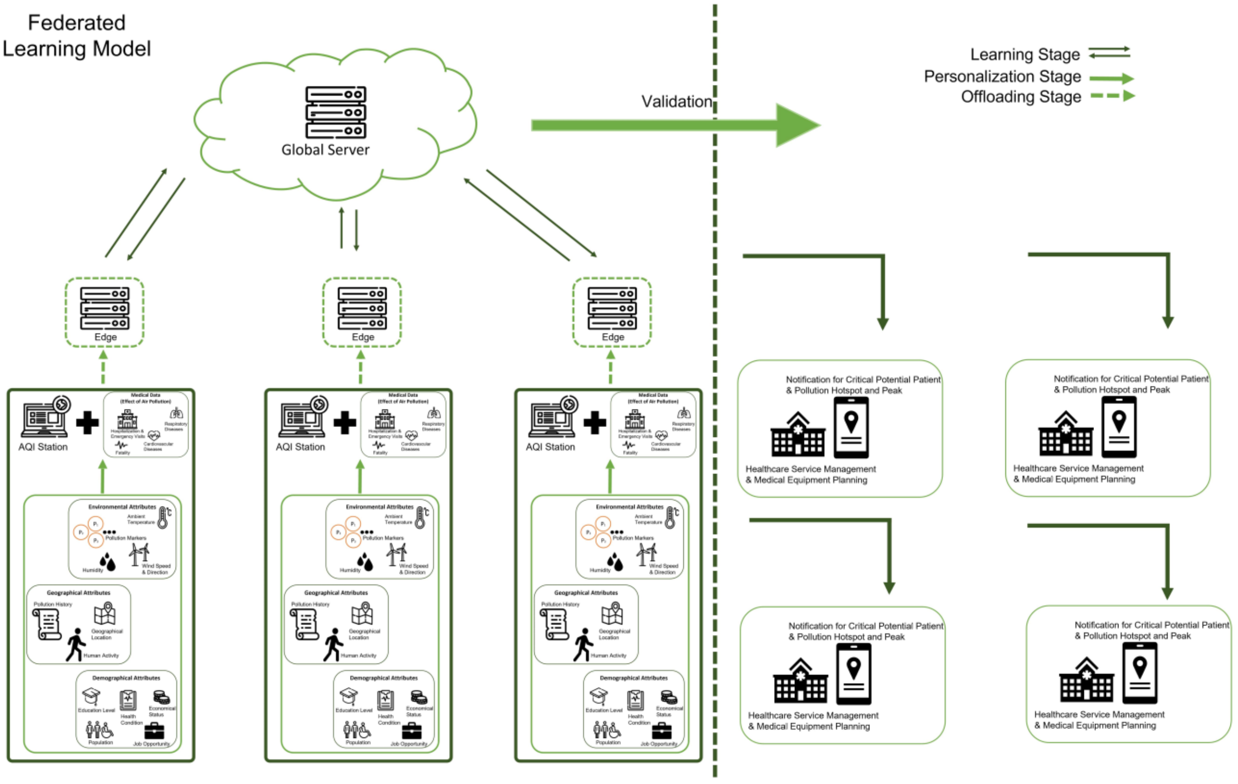

Figure 2 Framework of Integrated Environmental and Health Impact Assessment.

1.2 Supplementary Tables

Table 1 Search string of databases (Scopus and Web of Science).

Database	Search String
**Scopus**	(TITLE-ABS-KEY ("air quality") AND TITLE-ABS-KEY ("machine learning") AND TITLE-ABS-KEY ("health"))
**Web of Science**	("air quality") AND TITLE-ABS-KEY ("machine learning") AND TITLE-ABS-KEY ("health")

Table 2 Inclusion and exclusion criteria for the searching in databases.

Criterion	Inclusion	Exclusion
**Literature Type**	Journal (research article)	Journal (review articles), conference proceeding, book series, book chapter, book, encyclopedia
**Language**	English	Non-English
**Timeline**	2010-2021	< 2010
**Area**	Engineering, Environmental Science, Health Science, Artificial Intelligence	Other than Engineering, Environmental Science, Health Science, Artificial Intelligence

Table 3 Search Strings for 7 Databases.

Searching Texts	Science Direct	IEEE Xplore	Web of Science	SAGE	Emerald	Dimensions	Scopus
**Air pollution** AND telehealth	12	0	7	0	0	395	50
**Air quality** AND telehealth	11	0	2	2	0	335	41
**Air pollution** AND digital health	9	0	6	0	10	364	2
**Air quality** AND digital health	10	0	0	1	9	310	71
Sustainable health AND **Air quality**	51	0	1	1	8	282	77
Sustainable health AND **Air pollution**	35	0	7	1	6	350	82
**Air quality** AND **Machine learning** AND **Health**	1407	14	168	18	60	7390	2000

**Air quality** AND **Deep Learning** AND **Health**	451	8	74	5	23	2864	94
**Total including duplicates**	1986	22	265	28	116	12290	2417
**Sub-total including duplicates**	17124						
**Total selected**	18						

Table 4 Summary and overview of the review findings.

Authors	Air pollution markers	Health hazard impact from air pollution	Techniques	
			Prediction/ Monitoring Model	Association Assessment
Reid, Considine (2) *(United States (US))*	Particulate Matter ($PM_{2.5}$), Ozone (O_3)	Respiratory diseases	Generalized additive model (GAM), generalized boosting model (GBM), k-nearest neighbor model regression, lasso regression,	Poisson generalized estimating equations
Usmani, Pillai (3) *(Malaysia)*	Particulate Matters (PM_{10}), Ozone (O_3), carbon monoxide (CO), nitrogen oxides (NO_x), nitrogen dioxides (NO_2), nitrogen monoxide (NO), sulphur dioxide (SO_2)	Cardiorespiratory diseases	Enhanced long short-term memory (ELSTM)	-
Tusnio, Fichna (4) *(5)*	Sulphur dioxide (SO_2), nitrogen dioxides (NO_2), nitrogen oxides	Various types of cancers	Random forest	Pearson correlation coefficient

	(NO_x), carbon monoxide (CO),Ozone (O_3), Particulate Matters ($PM_{2.5}$,PM_{10}), benzene (C_6H_6), Lead (Pb), Arsenic (As), Cadmium (Cd), Nickel (Ni), Benzo(a)pyrene (BaP) in PM_{10} size			
Wang, Li (6) *(China)*	Particulate Matters ($PM_{2.5}$, and PM_1)	Blood cell counts for pregnancy preparation	-	Generalized additive mixed model (GAMM)
Achebak, Petetin (7) *(Spanish)*	Ozone (O_3), nitrogen monoxide (NO),	Premature mortality	-	Quasi-Poison regression model
Wang, Li (8) *(China)*	Particulate Matters ($PM_{2.5}$, and PM_1)	Blood Pressure	Random forest model	Generalized additive mixed model (GAMM)
Zani, Lonati (9) *(Equatorial Asia)*	Particulate Matters ($PM_{2.5}$, and PM_{10})	Premature Mortality	Deep neural network (DNN)	Generalized exposure mortality mixed model
Zou, O'Neill (10) (*Western U.S, Pacific Northwest (PNW))*	Particulate Matters ($PM_{2.5}$)	Mortality	Ordinary multi-linear regression, generalized boosting method, random forest	-
Cazzolla Gatti, Velichevskaya (11) *(Italy)*	Particulate Matters ($PM_{2.5}$ and PM_{10}), nitrogen dioxide (NO_2), sulphur dioxide (SO_2), carbon monoxide	Mortality and infectivity of COVID-19	Random forest regression, Pearson's correlation coefficient	-

	(CO), Benzene (C6H6), ozone (O_3)			
Sethi and Mittal (12) *(India, Delhi)*	Carbon monoxide (CO), sulphur dioxide (SO_2), particulate matters ($PM_{2.5}$), Ozone (O_3), nitrogen dioxide (NO_2), ammonia (NH_3), toluene (C_7H_8), benzene (C_6H_6)	Covid-19 fatalities	Decision tree, linear regression, random forest	-
Peng, Chen (13) *(China)*	Carbon monoxide (CO), sulphur dioxide (SO_2), nitrogen dioxide (NO_2), ozone (O_3), particulate matters ($PM_{<2.5}$)	Respiratory Disease	Bagging, adaptive boosting, and random forest	-
Shen, Valagolam (14) *(South Korea)*	Particulate matter ($PM_{2.5}$ and PM_{10}), carbon monoxide (CO), nitrogen dioxide (NO_2), sulphur dioxide (SO_2)	-	Prophet forecasting model (PFM)	-
Al Noaimi, Yunis (15) (*Lebanese Republic)*	Particulate Matters ($PM_{2.5}$), sulphur dioxide (SO_2), nitrogen dioxide (NO_2)	Prenatal and birth defect	Multivariate regression models	-
Li, Jing (16) *(China)*	Particulate Matters ($PM_{2.5}$)	Esophageal cancer	-	quasi-Poisson generalized linear model
Amoroso, Cilli (17)	carbon monoxide (CO), nitrogen dioxide (NO_2), ozone (O_3),	Covid-19 mortality	Random forest	-

(*European Countries)*	methane (CH_4), formaldehyde (CH_2O), aerosol			
Hadei, Hopke (18) *(Iran)*	Particulate Matters ($PM_{2.5}$ and PM_{10}), nitrogen dioxide (NO_2), ozone (O_3),	Covid-19 mortality and morbidity	Distributed-lag non-linear model (DLNM), generalized additive model (GAM)	-
Li, Guo (19) *(China)*	Particulate Matters ($PM_{2.5}$)	Esophageal cancer	-	Geographic weighted Poisson Regression
Ren, Zhu (20) *(China)*	Particulate Matters (PM_{10})	Congenital heart defects	Random forest (RF) and gradient boosting (GB)	-

Table 5 Recommendation of Air Quality Index (AQI) classification by US EPA (21).

**AQI Color**	**Index Value**	**Pollution Level of Concern**	**AQI Description**
**Green**	0-50	Good	Air pollution and satisfactory air quality pose little or no harm.
**Yellow**	51-100	Moderate	Air quality is adequate. Some people, however, may be at danger, particularly those who are highly sensitive to air pollution.
**Orange**	101-150	Unhealthy for sensitivity groups	Members of the sensitive group may suffer health consequences. Less liked to have an impact on the broader populace.
**Red**	151-200	Unhealthy	Some members of the general population may suffer from health

			consequences, while members of sensitive groups may suffer from more significant health problems.
**Purple**	201-300	Very unhealthy	Health warning: Everyone is at elevated risk of adverse health impacts.
**Maroon**	≥301	Hazardous	Everyone is most likely to be impacted by emergency situations, according to a health warning.

Table 6 Recommendation of pollutant concentrations by National Ambient Air Quality Standards (NAAQS) by US. EPA (22).

**Pollutant**		**Average Time of Exposure**	**Level of Exposure**
**Carbon Monoxide (CO)**		8 hours	9 ppm
		1 hour	35 ppm
**Lead (23)**		3 months average	0.15 ug/m^3
**Nitrogen Dioxide (NO_2)**		1 hour	100 ppb
		1 year	53 ppb
**Ozone (O_3)**		8 hours	0.070 ppm
**Particulate Matters / Particles Pollutions**	$PM_{2.5}$	1 year	12.0 ug/m^3
		1 year	15.0 ug/m^3
		24 hours	35 ug/m^3
	PM_{10}	24 hours	150 ug/m^3

Table 7 Summary of Air Quality Guidelines (AQG) levels and interim targets recommendations by WHO. (24)

Pollutant	Average Time	Interim Target 1 (IT-1) μg/m³	Interim Target 2 (IT-2) μg/m³	Interim Target 3 (IT-3) μg/m³	Interim Target 4 (IT-4) μg/m³	Air Quality Guidelines Levels μg/m³
**$PM_{2.5}$**	Annual	35	25	15	10	5
	24-hours	75	50	37.5	25	15
**PM_{10}**	Annual	70	50	30	20	15
	24-hours	150	100	75	50	45
**O_3**	Peak Season	100	70	-	-	60
	8-hours	160	120	-	-	100
**NO_2**	Annual	40	30	20	-	10
	24-hours	120	50	-	-	25
**SO_2**	24-hours	125	50	-	-	40
**CO*** ***in mg/m³**	24-hours	7	-	-	-	4

Table 8 Air Pollution Index classification recommended by Department of Environment (DoE), Malaysia (25).

Air Pollution Index (API)	API Status	Color	API Description

**0-50**	Good	Blue	There is little pollution, and these are no harmful health effects.
**51-100**	Moderate	Green	It has no harmful effects on health.
**101-200**	Unhealthy	Yellow	Sensitive folks should avoid. Health conditions for the elderly, pregnant women, children, and persons with heart and lung issues deteriorate.
**201-300**	Very Unhealthy	Orange	Unhealthy for the public. Worsening health and a reduced tolerance for physical activity might lead to lungs and heart issues.
**>301**	Hazardous	Red	Emergency

Table 9 Ambient Air Quality Standard in Malaysia by Department of Environment (DoE), Malaysia(26).

**Pollutant**	**Averaging Time**	**Ambient Air Quality Standard**
**PM_{10}**	Annually	40 µg/m^3
	24 Hours	100 µg/m^3
**$PM_{2.5}$**	Annually	15 µg/m^3
	24 Hours	35 µg/m^3
**SO_2**	1 Hour	250 µg/m^3
	24 Hours	80 µg/m^3
**NO_2**	1 Hour	280 µg/m^3

	24 Hours	70 µg/m^3
**O_3**	1 Hour	180 µg/m^3
	24 Hours	100 µg/m^3
**CO**	1 Hour	30 mg/m^3
	24 Hours	10 mg/m^3

1. Page MJ, McKenzie JE, Bossuyt PM, Boutron I, Hoffmann TC, Mulrow CD, et al. The PRISMA 2020 statement: an updated guideline for reporting systematic reviews. BMJ. 2021;372:n71.

2. Reid CE, Considine EM, Watson GL, Telesca D, Pfister GG, Jerrett M. Associations between respiratory health and ozone and fine particulate matter during a wildfire event. Environment International. 2019;129:291-8.

3. Usmani RSA, Pillai TR, Hashem IAT, Marjani M, Shaharudin R, Latif MT. Air pollution and cardiorespiratory hospitalization, predictive modeling, and analysis using artificial intelligence techniques. Environmental Science and Pollution Research. 2021;28(40):56759-71.

4. Tusnio N, Fichna J, Nowakowski P, Tofilo P. Air Pollution Associates with Cancer Incidences in Poland. Applied Sciences-Basel. 2020;10(21):13.

5. Maguire K, Garside R, Poland J, Fleming LE, Alcock I, Taylor T, et al. Public involvement in research about environmental change and health: A case study. Health (London, England : 1997). 2019;23(2):215-33.

6. Wang YY, Li Q, Guo YM, Zhou H, Wang QM, Shen HP, et al. Association between air particulate matter pollution and blood cell counts of women preparing for pregnancy: Baseline analysis of a national birth cohort in China. Environmental Research. 2021;200:8.

7. Achebak H, Petetin H, Quijal-Zamorano M, Bowdalo D, Pérez García-Pando C, Ballester J. Trade-offs between short-term mortality attributable to NO2 and O3 changes during the COVID-19 lockdown across major Spanish cities. Environmental Pollution. 2021;286:117220.

8. Wang Y-Y, Li Q, Guo Y, Zhou H, Wang Q-M, Shen H-P, et al. Long-term exposure to airborne particulate matter of 1 μm or less and blood pressure in healthy young adults: A national study with 1.2 million pregnancy planners. Environmental Research. 2020;184:109113.

9. Zani NB, Lonati G, Mead MI, Latif MT, Crippa P. Long-term satellite-based estimates of air quality and premature mortality in Equatorial Asia through deep neural networks. Environmental Research Letters. 2020;15(10):11.

10. Zou YF, O'Neill SM, Larkin NK, Alvarado EC, Solomon R, Mass C, et al. Machine Learning-Based Integration of High-Resolution Wildfire Smoke Simulations and Observations for Regional Health Impact Assessment. International Journal of Environmental Research and Public Health. 2019;16(12):20.

11. Cazzolla Gatti R, Velichevskaya A, Tateo A, Amoroso N, Monaco A. Machine learning reveals that prolonged exposure to air pollution is associated with SARS-CoV-2 mortality and infectivity in Italy. Environmental Pollution. 2020;267:115471.

12. Sethi JK, Mittal M. Monitoring the Impact of Air Quality on the COVID-19 Fatalities in Delhi, India: Using Machine Learning Techniques. Disaster Med Public Health Prep. 2020:1-8.

13. Peng JF, Chen C, Zhou M, Xie XH, Zhou YQ, Luo CH. Peak Outpatient and Emergency Department Visit Forecasting for Patients With Chronic Respiratory Diseases Using Machine Learning Methods: Retrospective Cohort Study. Jmir Medical Informatics. 2020;8(3):8.

14. Shen J, Valagolam D, McCalla S. Prophet forecasting model: a machine learning approach to predict the concentration of air pollutants (PM2.5, PM10, O-3, NO2, SO2, CO) in Seoul, South Korea. Peerj. 2020;8:18.

15. Al Noaimi G, Yunis K, El Asmar K, Abu Salem FK, Afif C, Ghandour LA, et al. Prenatal exposure to criteria air pollutants and associations with congenital anomalies: A Lebanese national study. Environmental Pollution. 2021;281:117022.

16. Li P, Jing J, Guo W, Guo X, Hu W, Qi X, et al. The associations of air pollution and socioeconomic factors with esophageal cancer in China based on a spatiotemporal analysis. Environmental Research. 2021;196:110415.

17. Amoroso N, Cilli R, Maggipinto T, Monaco A, Tangaro S, Bellotti R. Satellite data and machine learning reveal a significant correlation between NO2 and COVID-19 mortality. Environmental Research. 2022;204:10.

18. Hadei M, Hopke PK, Shahsavani A, Raeisi A, Jafari AJ, Yarahmadi M, et al. Effect of short-term exposure to air pollution on COVID-19 mortality and morbidity in Iranian cities. Journal of Environmental Health Science and Engineering. 2021:10.

19. Li P, Guo XY, Jing J, Hu WB, Wei WQ, Qi X, et al. The lag effect of exposure to PM2.5 on esophageal cancer in urban-rural areas across China. Environmental Science and Pollution Research. 2021:11.

20. Ren Z, Zhu J, Gao Y, Yin Q, Hu M, Dai L, et al. Maternal exposure to ambient PM10 during pregnancy increases the risk of congenital heart defects: Evidence from machine learning models. Science of The Total Environment. 2018;630:1-10.

21. AirNow. Air Quality Index (AQI) Basics [Available from: https://www.airnow.gov/aqi/aqi-basics/.

22. US. EPA. Criteria Air Pollutants - NAAQS Table [Available from: https://www.epa.gov/criteria-air-pollutants/naaqs-table.

23. Feinberg SN, Williams R, Hagler G, Low J, Smith L, Brown R, et al. Examining spatiotemporal variability of urban particulate matter and application of high-time resolution data from a network of low-cost air pollution sensors. Atmospheric Environment. 2019;213:579-84.

24. World Health Organization. Air Quality Guidelines - Second Edition 2000.

25. Environment Do. Air Pollution Index (API) [Available from: https://www.doe.gov.my/portalv1/en/info-umum/english-air-pollutant-index-api/100.

26. Department of Environment. Air Quality Standard [
